# Data-Driven Cluster Analysis of Oxidative Stress Indexes in relation to Vitamin D Level, Age, and Metabolic Control in Patients with Type 2 Diabetes on Metformin Therapy

**DOI:** 10.1155/2021/7942716

**Published:** 2021-06-21

**Authors:** Milena M. Cojic, Aleksandra Klisic, Radivoj Kocic, Andrej Veljkovic, Gordana Kocic

**Affiliations:** ^1^Primary Health Care Center, University of Montenegro, Faculty of Medicine, Podgorica, Montenegro; ^2^Clinic for Endocrinology, Faculty of Medicine, University of Nis, Nis, Serbia; ^3^Institute of Biochemistry, Faculty of Medicine, University of Nis, Nis, Serbia

## Abstract

Recent advances in vitamin D research indicate that patients with type 2 diabetes mellitus (T2DM) are suffering from vitamin D deficiency and increased oxidative stress to a variable extent, which could produce different health impacts for each individual. The novel multivariate statistical method applied in the present study allows metabolic phenotyping of T2DM individuals based on vitamin D status, metabolic control, and oxidative stress status in order to identify effectively different subtypes in our type 2 DM study population. Data-driven statistical cluster analysis was performed with 95 patients with T2DM, treated with metformin. Clusters were based on 12 variables—age, disease duration, vitamin D level, insulin, fasting glycemia (FG), glycated hemoglobin (HbA1c), high-density and low-density lipoprotein, total cholesterol (TC), triglycerides (TG), body mass index (BMI), and triglycerides/glucose index (TYG). The analysis revealed four unique clusters which differed significantly in terms of vitamin D status, with a mean 25 (OH) D level in cluster 1 (57.84 ± 11.46 nmol/L) and cluster 4 (53.78 ± 22.36 nmol/L), falling within the insufficiency range. Cluster 2 had the highest mean level of 25 (OH) D (84.55 ± 22.66 nmol/L), indicative of vitamin D sufficiency. Cluster 3 had a mean vitamin D level below 50 nmol/L (49.27 ± 16.95), which is considered deficient. Patients in the vitamin D sufficient cluster had a significantly better glycemic and metabolic control as well as a lower level of lipid peroxidation compared to other clusters. The patients from the vitamin D sufficient cluster also had a significantly higher level of vitamin D/MPO, vitamin D/XO, vitamin D/MDA, vitamin D/CAT, and vitamin D/TRC than that in the vitamin deficient and insufficient clusters. The vitamin D deficient cluster included significantly younger patients and had a significantly lower level of AOPP/TRC and albumin/TRC than the vitamin D sufficient cluster. The evidence from our cluster analysis in the context of separated T2DM demonstrates beneficial effects of optimal vitamin D status on metabolic control and oxidative stress in T2DM patients. Older T2DM patients require higher vitamin D levels in order to achieve good metabolic control and favorable antioxidant protection. Since protein damage is more pronounced in these patients, adding water-soluble antioxidant in addition to higher doses of vitamin D should be considered.

## 1. Introduction

Oxidative stress is defined as an imbalance caused by the increased production of free radicals and the inability of cellular antioxidant mechanisms to neutralize these products. It has been proposed as the root cause of the development of many chronic diseases. In type 2 diabetes mellitus (T2DM), oxidative stress plays an important role in the onset as well as in the progression of the disease [1, 2]. Chronic overnutrition and physical inactivity result in lipotoxicity and glucotoxicity, which cause the excessive formation of reactive oxygen species (ROS) and metabolic stress in various organs and tissues including pancreatic *β*-cells [3]. This is believed to be the main mechanism of the development of insulin resistance and altered insulin secretion. Due to decreasing beta-cell function, the individual with insulin resistance develops chronic hyperglycemia, which further promotes a prooxidant environment and production of ROS. Besides hyperglycemia, increased free fatty acids, leptin, and other circulating factors may also contribute to oxidative stress in T2DM. The increased ROS production in T2DM patients activates many detrimental pathways, including the inflammatory pathway involved in cell injury resulting in further disease progression and development of micro- and macrovascular complications [4, 5].

Some evidence suggests that vitamin D contributes to reduced oxidative stress by regulating several gene expressions involved in the antioxidant defense system. Furthermore, the antioxidant properties of vitamin D are thought to be more potent than those of vitamin E. Studies *in vitro* have confirmed this by showing that vitamin D inhibits lipid peroxidation and malondialdehyde (MDA) production to a greater extent than vitamin E analogues [6–8]. Cojic et al. pointed to a new potential mechanism of antioxidant and anti-inflammatory effect of vitamin D by showing that supplementation by vitamin D decreases the activity of myeloperoxidase (MPO), an enzyme involved in both ROS production and inflammation [2]. Nevertheless, many vitamin D supplementation trials in patients with T2DM have failed to show its favorable effect on oxidative stress [9].

It is unclear why even in the same clinical study vitamin D supplements affect only some of the oxidative stress outcomes tested. The effects of vitamin D supplementation on glycemic control in T2DM patients are controversial [8, 10]. There are a few possible reasons to explain why these trials failed to reach solid conclusions. First, most of the trials included very heterogeneous groups of patients with T2DM regarding gender, age, ethnicity, lifestyle, body mass index (BMI), metabolic control, and stage of the disease. Second, different doses of vitamin D were given in various manners, and many patients did not achieve adequate levels of vitamin D. Currently, there is no consensus on the optimal level for vitamin D status, but many scientists argue that levels ≥ 50 nmol/L are optimal only for skeletal health, while in order to achieve benefits for extraskeletal health, patients should have vitamin D levels between 75 and 125 nmol/L [11]. The third reason could be ascribed to the limitations in assessing oxidative stress in human subjects. There is a lack of evidence about which marker would be the most appropriate to evaluate *in vivo* oxidation [12].

To understand how exposure to the environment, diet, lifestyle, etc., interact with our unique characteristics and affect our health, the concept of the exposome was introduced as a measure of all internal and external environmental exposures [13]. Exposome is seen as complementary to the genome and represents all nongenetic factors that affect a human organism during its lifetime and whose interaction with the genome defines risk for disease development. Previous studies have been focused on a single or a limited number of exposures which provide only a fragmented view of exposome-health associations. Results from these studies may suffer from confounding due to unmeasured exposures. Applying a more holistic exposome approach can provide better insight into the relationship between internal and external stressors and health outcomes [14]. Patients with T2DM are affected by vitamin D insufficiency/deficiency to a variable extent, which can have different health impacts for each individual because of differences in genetic and other factors. One way to measure the biological response to complex exposure experience is through assessing metabolome, complete sets of biological molecules from metabolism, which can be directly studied for their impact on disease risk [13, 15]. Therefore, we studied the association between the internal exposome-baseline values of vitamin D and other internal exposomes such as lipidomics, diabetes, obesity, oxidative stress, and age. In this way, we were able to consider many exposure families simultaneously by applying a single statistical approach (a cluster analysis) and not separately as done in previous studies.

## 2. Materials and Methods

### 2.1. Design and Participants

A cross-sectional study was conducted in Montenegro, at Primary Health Care Center (PHCC) Podgorica, as a part of a previous study [2]. A total of 58 males and 51 females attending PHCC Podgorica from the 15^th^ of May until the 25^th^ of June 2018 were recruited to participate in the study. The inclusion criteria were both sexes, age ≥ 30 years, diagnosis of T2DM according to the American Diabetes Association (ADA) 2011 criteria for more than one year [16], treatment with metformin, and receipt of lifestyle advice. The exclusion criteria were the use of vitamin D supplements and any diabetes pharmacotherapy other than metformin, the use of steroids and/or anticonvulsants, the presence of severe anemia, chronic liver or kidney failure, alcoholism, pregnancy, malabsorption, and the presence of acute or chronic inflammatory conditions. The study was conducted in accordance with the approval of the Ethical Committee of PHCC in Podgorica (Ethical Committee of Primary Health Care Center in Podgorica, ID number 05/01-E.K.-5989/1), and all participants provided written informed consent.

### 2.2. Measurements

Body weight was measured to the nearest 0.1 kg with the participant dressed in light clothes and without shoes, using a Seca Alpha 770 digital scale. Body height was measured bare feet using a wall-mounted stadiometer with a mobile anthropometer (Seca 214, SECA Deutschland, Hamburg, Germany) to the nearest millimeter. BMI was calculated as weight in kilograms divided by the square of height in meters (kg/m^2^). Obesity was defined if BMI ≥ 30 kg/m^2^. Individuals were considered overweight if BMI was in the range of 25 to 29 kg/m^2^. Waist circumference (WC) was expressed in cm and measured while standing using the anthropometric tape positioned parallel to the floor over the umbilicus. Systolic (SBP) and diastolic (DBP) blood pressure measurement was performed twice, with a one-minute interval, and the average value was used. Measuring was done in a seated position, after a 10-minute rest, using an electronic monitoring device (Microlife's BP A150-30 AFIB).

### 2.3. Blood Samples and Analysis

Venous blood samples of the participants were collected early in the morning after 12 hours of overnight fasting. After the coagulation process was completed, all samples were centrifuged at 2000-3000 rpm for 10 min to separate the serum.

Standard biochemical parameters were performed within the Center for Laboratory Diagnostics of PHC Podgorica on the day of blood collection. The serum samples for oxidative stress analysis were divided into aliquots and stored at -80°C. Serum levels of fasting glucose (FG), creatinine, total cholesterol (TC), low-density lipoprotein (LDL) cholesterol, high-density lipoprotein (HDL) cholesterol, triglycerides (TG), albumin, aspartate aminotransferase (AST), alanine aminotransferase (ALT), *γ*-glutamyl transferase (GGT), and total calcium (Ca) were measured spectrophotometrically, using standard procedures, while glycated hemoglobin (HbA1c) was determined from full blood immunoturbidimetrically. The serum C reactive protein (CRP) level was also determined by the immunoturbidimetric method. All analyses were performed on Roche Cobas 6000, c 501, Mannheim, Germany. Ionized calcium (Ca++) was determined using the ion-selective method on an electrolyte analyzer (Roche Diagnostics AVL 9180 Electrolyte Analyzer (AVL 9180, Roche, Japan)). An electrochemiluminescence method was used to measure insulin and vitamin D level.

Vitamin D status was assessed based on determining the serum 25 (OH) D level. According to the Endocrine Society guidelines, patients were considered vitamin D deficient if their 25 (OH) D levels were ≤50 nmol/L. Levels between 50 and 75 nmol/L were considered insufficient, and the optimal vitamin D status was reflected by 25 (OH) D levels greater than 75 nmol/L [10].

The fasting homeostasis model assessment of insulin resistance (HOMA-IR) was calculated as a surrogate marker for insulin resistance assessment according to the formula: glucose (mmol/L) × insulin (*μ*IU/L)/22.5 [17] and TG glucose (TYG) index using the following formula: Ln [fasting TG (mg/dL) × fasting glucose (mg/dL)/2] [18].

### 2.4. Oxidative Stress Parameters

The MDA level was measured as a marker of oxidative lipid injury and expressed as the thiobarbituric acid-reactive substances (TBARS) [19]. We used the TG/TBARS index to monitor the ratio between the potentially oxidizable substrates, i.e., circulating triglycerides and their oxidized counterparts-lipid peroxides.

Advanced oxidation protein product levels (AOPP) were used to assess protein oxidative damage. In brief, AOPP were measured in serum spectrophotometrically and expressed as chloramine-T equivalents. Prior to the analysis, the samples were diluted in a 1 : 10 ratio [20].

Serum MPO activity was determined using a commercial ELISA test, as described [2]. The enzyme concentration was expressed as ng/L.

Serum xanthine oxidase (XO) was determined spectrophotometrically, as described [2]. The enzyme activity was based according to the uric acid liberation by using xanthine as a substrate. The enzyme activity was expressed as U/L.

Serum catalase (CAT) activity was obtained by using a spectrophotometric method, as described [2]. The enzyme activity was based on the rapid degradation of hydrogen peroxide from its stable yellow complex with ammonium molybdate. The enzyme activity was expressed as cat/L.

The concentration of nitrite and nitrate (commonly named NOx) in serum was used as an indicator of nitric oxide (NO) production and was determined in the following manner: the reduction of nitrate to nitrite was achieved using copper-coated cadmium. The measurement of nitrite concentration was performed by colorimetric detection of azo dye products of the Griess reaction [21].

Total oxidative capacity (TOC) was determined using the method based on the oxidation of the ferrous ion to ferric ion. The main components of TOC are hydrogen-peroxide (H_2_O_2_) and other peroxides that oxidize ferric ions. In an acidic environment, ferric ion forms a colored complex with xylenol-orange. The intensity of staining is determined spectrophotometrically, and it is directly proportional to the total number of oxidation molecules (peroxides) in the sample. The results are expressed as micromoles of H_2_O_2_ equivalents per liter (*μ*mol/L) [22].

The total reductive capacity (TRC) was determined using the Folin-Ciocalteu method with a small modification. The method is based on the oxidation of the phenols present with Folin's reagent, whereby the reagent is reduced. Gallic acid was used as a standard, and the results are presented as gallic acid equivalents (GAE) [23].

In order to avoid the bias of each marker measurement, we evaluated the ratios between different parameters. The degree of oxidative stress was evaluated by the oxidant/oxidized component and the antioxidant ratio. In order to gain insight into the pathophysiological processes of oxidative stress and the possible role of vitamin D, we calculated several ratios between various prooxidant and antioxidant parameters, such as MDA/TRC, MDA/TOC, TG/TBARS, AOPP/TRC, AOPP/TOC, AOPP/albumin, NOx/CAT, albumin/TRC, albumin/TOC, TOC/TRC, and vitamin D and oxidative biomarker ratios in relation to cluster analysis.

### 2.5. Statistical Analysis

Data-driven cluster analysis (agglomerative hierarchical clustering) was performed in patients with T2DM. Hierarchical cluster analysis starts from the point that every value is a cluster. Clusters are merged based on a similarity between individual values up to the highest cluster. In this cluster analysis, the distance between subjects was calculated using squared-Euclidian distance. This unsupervised cluster analysis of continuous variables included age, disease duration, vitamin D level, insulin, glycemia, HbA1c, HDL, LDL, TC, TG, BMI, and TYG. The Ward method with standardization of included variables was performed in the R programming language [24]. This method was performed because it minimizes the differences between individual values within clusters and maximizes the differences between formed clusters [25]. A dendrogram was generated to visualize clusters and to determine how many clusters to retain in the analysis. The dendrogram generates the network structure of the dataset, computing the Euclidian distance between each pair of individuals.

Significant determinants of cluster membership were identified by discriminant analysis. Once four clusters were generated, characteristics of clusters were compared using the following tests: analysis of variance (ANOVA) or *t*-test for normally distributed continuous variables or Kruskal-Wallis test and Mann-Whitney test for nonnormally distributed variables. Chi-squared analysis was used for categorical data. Data are presented as the mean ± standard deviation or median (25^th^–75^th^ percentile).

## 3. Results

Out of 109 patients, in the initial cluster analysis, we identified two subjects with outlying values of total cholesterol and triglycerides. After the exclusion of two outlying values and patients with incomplete clinical and biochemical profiles, the study was conducted on 95 T2DM patients. The demographic and clinical characteristics of subjects included in the study are shown in Table 1.

Discriminant analysis obtained that all variables were significant determinants in cluster membership. Reclassification revealed that the discriminant function model achieved 81% accuracy for predicting cluster membership. The TC level was the most significant determinant of cluster membership (*F* = 30.320, *p* < 0.001), followed by the TYG index (*F* = 22.846, *p* < 0.001), LDL level (*F* = 19.275, *p* < 0.001), and vitamin D level (*F* = 15.663, *p* < 0.001). Less significant were the TG level (*F* = 13.405, *p* < 0.001), FG (*F* = 10.961, *p* < 0.001), disease duration (*F* = 9.732, *p* < 0.001), HbA1c (*F* = 9.357, *p* < 0.001), insulin (*F* = 7.780, *p* < 0.001), HDL (*F* = 6.573, *p* < 0.001), age (*F* = 5.605, *p* < 0.001), and BMI (*F* = 5.055, *p* < 0.001).

Cluster 1 included the oldest patients who had the longest duration of the disease, the poorest glycemic control (the highest mean levels of FG, eAG, and HbA1c), the lowest mean level of HDL, and relatively high levels of insulin resistance and BMI.

Cluster 2 included elderly patients with optimal vitamin D status, good glycemic control (the lowest levels of eAG and HbA1c), low insulin resistance (the lowest levels of insulin, HOMA-IR, and TYG index), the highest mean level of HDL, and lowest mean levels of TG and BMI (Table 2).

Cluster 3 included young middle-aged patients, with the shortest disease duration, the poorest vitamin D status, good glycemic control (the lowest mean level of FG, low HbA1c), relatively high insulin resistance, and good lipid status (the lowest levels of TC and LDL). These patients were obese with the highest mean level of BMI (Table 2).

Cluster 4 included middle-aged, overweight patients, with shorter disease duration, the highest levels of insulin resistance markers (the highest mean levels of insulin, HOMA-IR, and TYG index), the poorest lipid profile (the highest mean levels of TC, TG, and LDL), increased FG mean level, and moderately high HbA1c mean level (6.5 ≤ HbA1c ≤ 7%).

**Figure 1:**
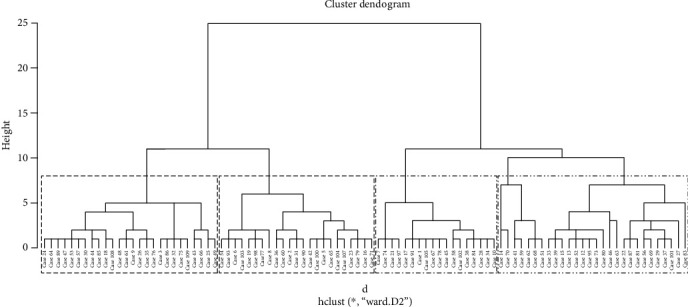
Dendrogram of clustering age, disease duration, vitamin D level, insulin, glycemia, HbA1c, HDL, LDL, TC, TG, BMI, and TYG.

The analysis revealed four unique clusters of patients with T2DM (Figure 1), which differed significantly in terms of vitamin D status, with a mean 25 (OH) D level in cluster 1 (57.84 ± 11.46 nmol/L) and cluster 4 (53.78 ± 22.36 nmol/L) falling within insufficiency range. Cluster 2 had the highest mean level of 25 (OH) D (84.55 ± 22.66 nmol/L) that was indicative for vitamin D sufficiency. Cluster 3 had a mean vitamin D level below 50 nmol/L (49.27 ± 16.95) which is considered deficient. Clusters also differed regarding age (*p* < 0.001), disease duration (*p* < 0.001), vitamin D level (*p* < 0.001), insulin level (*p* < 0.001), HOMA-IR index (*p* = 0.001), FG (*p* < 0.001), HbA1c (*p* < 0.001), average glucose levels (aAG) (*p* < 0.001), TC levels (*p* < 0.001), TG levels (*p* < 0.001), HDL-c levels (*p* = 0.003), LDL-c levels (*p* < 0.001), AST levels (*p* = 0.004), GGT levels (*p* = 0.025), albumin levels (*p* = 0.010), BMI (*p* = 0.036), and TYG index (*p* < 0.001).

When comparing oxidative stress indicators, it revealed that patients in clusters differed significantly regarding levels of MDA (*p* = 0.008), AOPP/TRC (*p* = 0.026), albumin/TRC (*p* = 0.024), and TG/TBARS (*p* = 0.002) (Table 3). Cluster 2 had significantly lower levels of MDA than cluster 4 (*p* < 0.001) and significantly lower TG level. Cluster 3 had significantly lower levels of AOPP/TRC than cluster 1 (*p* = 0.010) and cluster 2 (*p* = 0.041). Cluster 4 had significantly lower levels of AOPP/TRC than cluster 1 (*p* = 0.031). Cluster 3 had significantly lower levels of albumin/TRC than cluster 1 (*p* = 0.006) and cluster 2 (*p* = 0.0024). Cluster 1 had significantly higher levels of TG/TBARS compared to cluster 2 (*p* = 0.036) and cluster 3 (*p* = 0.002). Cluster 4 had significantly higher levels of TG/TBARS compared to cluster 2 (*p* = 0.017) and cluster 3 (*p* = 0.002).

Furthermore, as shown in Table 4, cluster 2 had a significantly higher level of vitamin D/MPO (*p* = 0.011, *p* = 0.002), vitamin D/XO (*p* = 0.001, *p* = 0.001), vitamin D/MDA (*p* < 0.001, *p* < 0.001), vitamin D/CAT (*p* < 0.001, *p* < 0.001), vitamin D/TRC (*p* < 0.001, *p* < 0.001), and vitamin D/GGT (*p* < 0.001, *p* < 0.001) than clusters 3 and 4. Two vitamin D insufficient clusters (clusters 1 and 4) differed significantly concerning vitamin D/MDA (*p* = 0.020) and vitamin D/GGT (*p* = 0.048). Cluster 2 had significantly higher levels of vitamin D/XO (*p* = 0.029), vitamin D/MDA (*p* = 0.001), vitamin D/CAT (*p* < 0.001), vitamin D/GGT (*p* = 0.002), and vitamin D/albumin (*p* < 0.001) than cluster 1. Cluster 2 had a significantly higher level of vitamin D/NOx (*p* = 0.003) and vitamin D/albumin than cluster 4. Cluster 1 had a significantly higher level of vitamin D/TRC (*p* < 0.001), but a significantly lower level of TRC/albumin (*p* = 0.010). Figure 1 represents a dendrogram of clustering using the Ward method and standardized scores (age, disease duration, vitamin D level, insulin, glycemia, HbA1c, HDL, LDL, TC, TG, BMI, and TYG, included in cluster analysis).

## 4. Discussion

In the present study, we first tried to evaluate whether the patients with diabetes could be classified according to internal baseline values of vitamin D and other internal exposomes and, after, check whether parameters of oxidative stress could be associated with this classification. Therefore, unsupervised clustering of all patients based on vitamin D, demographic variables, parameters of glycemic control, and lipid status resulted in optimally four clusters or phenotypes. This approach has been recently used to capture the diverse pathophysiological pathways and to classify distinct entities with different disease trajectories or differential responses to treatment in different medical areas [26–29]. Clustering was used in oxidative stress background successfully in dyslipidemia [30] and critically ill patients [31].

In addition, the multidimensional nature of antidiabetic treatment led to the application of a clustering method for the classification of T2DM patients and their association with oxidative stress parameters.

The application of a cluster analytical approach to a well-characterized cohort of adults with T2DM showed that vitamin D status is an important determinant of the clinical phenotype of T2DM. The cluster with the highest level of vitamin D had a significantly lower insulin resistance than vitamin D deficient or insufficient clusters, but a significantly better glycemic control compared to the vitamin D insufficient cluster. The vitamin D deficient cluster included significantly younger patients with a shorter disease duration than the vitamin D sufficient cluster, and that could be the reason for not having a more prominent difference in glycemic control. This could also mean that patients' age is a decisive factor for optimal metabolic control and that older patients might require higher levels of vitamin D in order to achieve target levels of glycemia and HbA1c. All patients used metformin, which could have masked small but significant differences between vitamin D sufficient and deficient clusters. Although there is strong evidence from observational studies that vitamin D is correlated with glycemic control in T2DM patients, there is still insufficient data to explain the role of vitamin D in treating these patients [32]. There are many reasons why interventional studies failed to provide consistent results. One of them is the lack of consensus on optimal vitamin D status. The Endocrine Society guidelines claim that extraskeletal effects are only achieved when vitamin D levels reach levels above 75 nmol/L [11]. Regarding oxidative stress markers, our study demonstrated that patients in the cluster with optimal vitamin D levels had a significantly lower level of lipid peroxidation than patients in clusters with vitamin D insufficiency. Our results also showed that patients in the vitamin D deficient cluster had a significantly lower level of lipid peroxidation than patients in the vitamin D insufficient clusters. Lipid peroxidation levels were measured as TG/TBARS.

Research has so far showed that oxidative stress plays a pivotal role in diabetes pathogenesis although exact mechanisms remained still unknown [33]. Oxidative degradation of lipids has been closely associated with chronic hyperglycemia in T2DM which promotes free radical production. Furthermore, the lipid structure of the cell membrane makes them more susceptible to damage caused by free radicals [34]. Some studies have shown that even well-controlled patients with T2DM have increased levels of lipid peroxidation and systemic inflammation [35]. There are several pieces of evidence to support the antioxidant role of vitamin D through a mechanism involved in reducing the plasma level of MDA. It is believed that this protective effect of vitamin D on cell membranes directly occurs through its hydrophobic parts [36]. There are only a few studies examining the relation between vitamin D and lipid peroxidation in patients with T2DM, one of which was cross-sectional and the others were interventional. The study conducted by Kumar et al. investigated the association of vitamin D deficiency with oxidative stress in newly diagnosed T2DM patients. The results showed that vitamin D levels were negatively correlated with MDA levels in newly diagnosed T2DM patients [37].

Intervention studies showed inconclusive results. Eftekhari et al. showed no significant reduction in MDA levels following 12 weeks of vitamin D supplementation in patients with unknown baseline vitamin D status, while Shab-Bidar et al. showed a significant reduction of MDA levels after the same period of intervention in patients with a baseline level of serum 25 (OH) D < 40 nmol/L [38, 39]. Significantly lower levels of TG/TBARS in the vitamin D deficient cluster could be explained by younger age and disease duration as well as by better glycemic and lipid control since these factors were found to influence oxidative stress in T2DM [40–42].

The evaluation of other individual markers of oxidative stress (oxidants, oxidized molecules, and antioxidants) in our study did not show a significant difference between clusters. These results are in agreement with other studies in which some oxidative stress markers were affected by vitamin D status and some not [34]. Bearing in mind the difficulty of measuring oxidative stress in humans regarding its complexity and the high number of oxidative stress parameters, we calculated the ratio between different oxidative stress markers [43] in order to overcome a bias of each measurement method.

The results from our study showed that the vitamin D sufficient cluster had a significantly lower TG/TBARS ratio. This is reasonable considering that cluster 2 showed a significantly lower TG level. It may be associated with the fact that besides endogenous synthesis, vitamin D-rich diet contains vegetables and fish, which have low TG levels. The vitamin D sufficient cluster had a significantly higher level of oxidative stress index measured as AOPP/TRC and also a significantly lower protective effect on albumin measured as albumin/TRC than the vitamin D deficient cluster. Specifically, the lack of beneficial effect of vitamin D on proteins could be explained by its lipophilic nature. Decreased albumin levels are associated with an unfavorable metabolic profile, characterized by increased adipose tissue inflammation, adiposity, and glycemia. This protein is providing > 50% of total antioxidant in normal plasma [44]. The mean albumin level was significantly lower in the cluster with the patients with higher BMI and vitamin D deficient status compared to the cluster with the overweight and vitamin D insufficient patients. Additionally, AOPP is derived mainly from oxidation-modified albumin and indicates not only oxidative stress but also the presence of coexisting inflammation [45]. The fact that the best protective effect on proteins was seen in the cluster with the younger patients could mean that older patients might benefit from the addition of water-soluble antioxidants in their therapy.

Recent evidence has suggested that none of the previously used biomarkers is ideal in evaluating oxidative stress in T2DM. The relative importance of each biomarker and its relation to the key mechanisms of the disease are still unknown [42]. In order to understand the role of vitamin D in complex modulating mechanisms of oxidative stress in T2DM, we calculated the ratio between vitamin D and different oxidative stress markers.

The results revealed similar effects on lipid peroxidation and oxidative protein damage, but ratios calculated provided insights into possible mechanisms through which vitamin D plays the key role in reducing oxidative stress in the vitamin D sufficient cluster. The vitamin D/MDA ratio confirmed a positive effect of vitamin D on lipid peroxidation in patients with T2DM and sufficient levels of vitamin D. Besides this lipoprotective effect, our results showed that vitamin D could have a beneficial anti-inflammatory and antioxidative effect on patients with T2DM measured as the vitamin D/MPO ratio because its mean level was significantly higher in vitamin D sufficient than in vitamin D insufficient and deficient clusters. This enzyme is a hemoprotein stored in azurophilic granules of polymorphonuclear neutrophils and macrophages and is secreted in inflammatory conditions. Together with hydrogen peroxide and chloride, it produces the powerful oxidant hypochlorous acid and it has been implicated in further tissue damage. It also plays an important role in atherogenesis by oxidatively modifying lipids containing LDL cholesterol, reducing NO bioavailability, and impairing its vasodilating and anti-inflammatory properties [46–48]. Although the literature regarding antioxidative effects of vitamin D remained scarce, Nikooyeh et al. demonstrated a significant improvement in the MPO activity in 90 T2DM patients supplemented with a 1000 IU fortified vitamin D yoghurt drink daily for four months [49]. Since this enzyme predicts endothelial function in humans and serves as a link among inflammation (activated leukocytes), oxidative stress, and endothelial dysfunction, our results demonstrated that adequate vitamin D status in patients with T2DM could protect against atherosclerosis [50]. Unexpectedly, patients from the vitamin D deficient cluster did not differ significantly from patients in the vitamin D insufficient cluster regarding the vitamin D/MPO ratio. Since the vitamin D deficient cluster included the youngest patients, these results could confirm previous findings that age is associated with dysregulated immune and inflammatory responses, which could be potentially improved by achieving better vitamin D status in older people [51].

Similarly, patients from the vitamin D sufficient cluster had the highest level of vitamin D/XO ratio meaning that vitamin D could inhibit the XO activity in patients with T2DM. Together with nicotinamide adenine dinucleotide phosphate (NADPH) oxidase and NO synthase, XO plays a physiologic role in inflammatory signaling, the regulation of NO production, and vascular function [52]. So far, research has shown that patients with T2DM have increased XO activity, which could be contributing to endothelial dysfunction, the main feature of diabetic macroangiopathy [53, 54]. To the best of our knowledge, there is little information about the effect of vitamin D on XO. The results from interventional trials showed that vitamin D supplementation improved endothelial function in patients with T2DM, but the exact mechanisms are only partly known [55, 56]. One of the plausible mechanisms could be mediated through XO activity. This enzyme suppresses genes involved in the expression of nuclear factor-*κ*B (NF-*κ*B). The inhibition of NF-*κ*B results in decreased expression of proinflammatory mediators which are involved in free radical production through upregulating XO activity [57]. Metformin use is one of the confounding factors that could explain why we did not get a significant difference in XO levels between clusters since it is known that oral therapy with metformin inhibits XO activity [58].

Results from our study also showed that the vitamin D sufficient cluster had the highest level of vitamin D/NOx ratio, but a significant difference was seen only compared to cluster 4 which included vitamin D insufficient patients with the highest mean level of insulin resistance. A meta-analysis aimed at examining NOx levels in patients with T1DM and T2DM showed that the levels of these nitrogen products were elevated, which supports the hypothesis that high NOx values are associated with adverse clinical events that accompany patients with diabetes [59]. Vitamin D is able to stimulate NO production by regulating the expression of inducible NO synthase [60]. Regulation of NO metabolism is particularly important in patients with T2DM, especially since NO synthase activation is controlled by insulin. In this way, insulin resistance impairs NO-mediated vasodilation and results in endothelial dysfunction [61]. Our results are in accordance with these findings because other clusters had only mildly elevated HOMA-IR levels in relation to cluster 2 and cluster 4, which could have contributed to better levels of the vitamin D/NOx ratio.

The damaging effects of oxidative stress are mainly modified by enzymatic or nonenzymatic antioxidants such as superoxide dismutase, CAT, glutathione peroxidase, minerals, vitamins, and polyphenols. Current evidence implicates that patients with T2DM have not only increased free radical production but also diminished the antioxidative defense system. Some experimental studies showed that vitamin D may influence antioxidant status by modifying some of the antioxidant enzymes [62, 63]. A meta-analysis examining the antioxidant properties of vitamin D showed that supplementation with this vitamin increased glutathione peroxidase activity and total antioxidant capacity, which is a measure of the synergistic effect of individual enzymatic and nonenzymatic antioxidants. Our results also showed that patients from the vitamin D sufficient cluster had significantly higher levels of vitamin D/TRC and vitamin D/CAT ratio meaning that optimal levels of vitamin D in patients with T2DM have a beneficial effect on antioxidant status [63, 64].

Finally, our study had some limitations. Because of its cross-sectional design, a causal relationship between vitamin D status and oxidative stress could not yet be established. We did not provide data about dietary habits and medical therapy, which could influence oxidative stress levels in the patients. The strength of our study is the novel cluster analysis according to their vitamin D status and metabolic phenotype, which enabled us to differentiate oxidative stress levels not only in vitamin D deficient and vitamin D sufficient patients but also in vitamin D insufficient patients. Our findings confirm the assumption that only levels greater than 75 nmol/L exert beneficial effects on glycemic control and oxidative stress. To our best knowledge, this is the first study to calculate the ratio between vitamin D and oxidative stress markers, which provided insight into plausible mechanisms of vitamin D antioxidative effects. Our findings suggest that the use of the aforementioned ratios may more accurately reflect the relationship between vitamin D and oxidative stress rather than oxidative stress markers by themselves.

## 5. Conclusions

The novel multivariate data-driven cluster analysis approach applied in the present study allows metabolic phenotyping of T2DM individuals and reveals beneficial effects of optimal vitamin D status on metabolic control and oxidative stress status in those patients through various pathways. The evidence from our study suggests that older patients with T2DM require higher vitamin D levels in order to achieve optimal metabolic control and favorable antioxidant protection. Since protein damage is more pronounced in these patients, adding water-soluble antioxidants in addition to higher doses of vitamin D should be considered.

Further trials are needed to establish the casual relationship between specific climate environmental factors, age, vitamin D status, metabolic control, and oxidative stress in order to determine health outcomes that vitamin D supplementation can produce in patients with T2DM.

## Figures and Tables

**Table 1:** Demographic and clinical characteristics of the subjects.

	Mean ± SD	Median	Minimum	Maximum
Age	61.01 ± 7.94	62.00	42.00	76.00
Gender (male/female) (*n*/%)	50/45	52.6/47.7		
T2DM duration (years)	6.01 ± 3.03	6.00	1.00	10.00
Vitamin D level (nmol/L)	60.76 ± 23.56	56.79	19.10	127.60
Insulin (*μ*U/L)	11.85 ± 5.87	10.41	2.01	28.22
HOMA-IR	4.17 ± 2.36	3.54	0.62	11.82
FG (mmol/L)	7.86 ± 1.6	7.70	5.30	15.50
HbA1c (%)	6.69 ± 0.93	6.50	4.90	9.40
eAG (mmol/L)	8.08 ± 1.49	7.80	5.20	12.40
Creatinine (*μ*mol/L)	82.03 ± 20.44	79.00	8.70	133.00
TC (mmol/L)	5.52 ± 1.27	5.28	3.09	9.24
TG (mmol/L)	1.83 ± 1	1.65	0.52	5.36
HDL-c (mmol/L)	1.32 ± 0.32	1.29	0.80	2.32
LDL-c (mmol/L)	3.44 ± 1.3	3.31	1.36	9.92
AST (IU/L)	20.04 ± 6.54	19.00	7.00	57.00
ALT (IU/L)	24.26 ± 17.49	20.00	6.00	162.00
ALP (IU/L)	62.36 ± 14.8	59.00	33.00	109.00
GGT (IU/L)	23.57 ± 13.15	19.00	6.00	77.00
Ca (mmol/L)	2.41 ± 0.11	2.20	2.20	2.82
Ca++ (mmol/L)	1.14 ± 0.07	1.13	0.93	1.35
CRP (mg/L)	2.78 ± 3.35	1.70	0.30	22.07
Albumin (g/L)	48.44 ± 2.31	48.00	44.00	55.00
BMI (kg/m^2^)	29.81 ± 4.59	29.07	17.30	46.60
SBP (mmHg)	137.96 ± 21.03	135.75	90.00	198.50
DBP (mmHg)	82.61 ± 10.99	80.50	60.00	111.50

**Table 2:** Basic demographic and clinical parameters in relation to cluster analysis.

Parameter	Cluster 1	Cluster 2	Cluster 3	Cluster 4	*p* ^1^
	*N* = 18	*N* = 23	*N* = 26	*N* = 28	
Age (years)	64.94 ± 7.32^b^	64.17 ± 6.23^b^	57.38 ± 6.83	59.25 ± 8.75	<0.001
Gender (M/F)	10/8	13/10	12/14	15/13	0.885^2^
T2DM duration (years)	8.64 ± 1.49^b,c^	6.72 ± 3.10^b,c^	4.6 ± 2.63	5.05 ± 2.88	<0.001^3^
Vitamin D level (nmol/L)	57.84 ± 11.46^a,b^	84.55 ± 22.66^b,c^	49.27 ± 16.95	53.78 ± 22.36	<0.001^3^
Insulin (*μ*U/L)	10.28 ± 4.69^c^	8.11 ± 3.59^b,c^	13 ± 5.47	14.86 ± 6.6	<0.001^3^
HOMA-IR	4.13 ± 1.84^a^	2.67 ± 1.23^b,c^	3.99 ± 1.82	5.59 ± 2.98	0.001^3^
FG (mmol/L)	9.13 ± 1.19^a,b,c^	7.39 ± 0.97	6.91 ± 0.87^c^	8.31 ± 2.07	<0.001^3^
HbA1c (%)	7.47 ± 0.82^a,b^	6.23 ± 0.58^c^	6.37 ± 0.79	6.87 ± 1.02	<0.001
eAG (mmol/L)	9.34 ± 1.33^a,b^	7.35 ± 0.93^c^	7.57 ± 1.27	8.36 ± 1.61	<0.001
Creatinine (*μ*mol/L)	85.61 ± 18.74	77.38 ± 19.76	80.15 ± 17.47	85.29 ± 24.32	0.802^3^
TC (mmol/L)	5.09 ± 1.05^c^	5.3 ± 0.82^c^	4.61 ± 0.82^c^	6.83 ± 0.99	<0.001
TG (mmol/L)	2.01 ± 0.67^a^	1.24 ± 0.49^c^	1.41 ± 0.66^c^	2.57 ± 1.25	<0.001^3^
HDL-c (mmol/L)	1.20 ± 0.30^a^	1.55 ± 0.39	1.29 ± 0.26^a^	1.22 ± 0.22^a^	0.003
LDL-c (mmol/L)	3.01 ± 0.92^c^	3.18 ± 0.78^c^	2.67 ± 0.75^c^	4.65 ± 1.45	<0.001^3^
AST (IU/L)	18 ± 4.07	20.7 ± 4.64	19.08 ± 3.75	21.68 ± 9.99	0.144^3^
ALT (IU/L)	18 ± 7.46^b,c^	21.09 ± 8.49^c^	21.81 ± 7.48^c^	33.18 ± 28.23	0.004^3^
ALP (IU/L)	62.39 ± 16.23	59.43 ± 11.9	65.19 ± 16.44	62.11 ± 14.72	0.645^3^
GGT (IU/L)	23.61 ± 14.55	18.73 ± 7.55^c^	21.73 ± 11.85^c^	29.07 ± 15.30	0.025^3^
Ca (mmol/L)	2.43 ± 0.12	12.05 ± 46.2	2.38 ± 0.13	2.41 ± 0.10	0.154
Ca++ (mmol/L)	1.15 ± 0.06	1.13 ± 0.06	1.14 ± 0.09	1.15 ± 0.06	0.645
CRP (mg/L)	3.02 ± 3.17	1.82 ± 1.84	3.82 ± 5.11	2.41 ± 1.8	0.418^3^
Albumin (g/L)	47.35 ± 2.34^c^	48.59 ± 1.84	47.73 ± 2.05^c^	49.62 ± 2.38	0.010
WC (cm)	104.19 ± 8.95	99.43 ± 10.04	107.17 ± 12.86	104.23 ± 8.74	0.253
BMI (kg/m^2^)	29.33 ± 3.01	27.55 ± 3.84	32.08 ± 5.72^a,c^	28.23 ± 6.32	0.036
SBP (mmHg)	138.19 ± 19.76	131.95 ± 22.15	134.23 ± 21.68	145.87 ± 19.01	0.062
DBP (mmHg)	80.5 ± 8.28	78.25 ± 8.54	83.98 ± 13.38	86.17 ± 10.96	0.097
TYG	9.54 ± 0.33^a,b^	8.82 ± 0.41^c^	8.86 ± 0.45^c^	9.92 ± 0.53	<0.001

^1^ANOVA; ^2^chi square; ^3^Kruskal-Wallis test; ^a^vs. cluster 2; ^b^vs. cluster 3; ^c^vs. cluster 4.

**Table 3:** Oxidative biomarkers and ratios in relation to cluster analysis.

	Cluster 1	Cluster 2	Cluster 3	Cluster 4	*p* ^1^
MPO (ng/L)^†^	104.52 (79.51-155.09)	112.45 (70.17-153.99)	92.01 (72.48-158.21)	105.7 (70.34-189.67)	0.787
XO (U/L)^†^	28.29 ± 9.69	28.35 ± 9.86	27.42 ± 8.77	30.21 ± 10.83	0.847^2^
AOPP (*μ*M chloramine-T equivalents)^†^	121.08 (100.53-318.16)	136.98 (89.03-383.53)	120.59 (93.44-186.69)	114.96 (101.14-133.67)	0.646
MDA (*μ*M/L)^†^	3.28 (2.47-3.79)	2.62 (2.41-3.05)^c^	3.15 (2.36-4.62)	3.63 (3.12-5.18)	0.008
CAT (cat/L)^†^	0.88 ± 0.07	0.87 ± 0.05	0.84 ± 0.08	0.86 ± 0.07	0.225^2^
NOx (*μ*mol/L)^†^	50.13 (36.36-65.91)	58.69 (48.47-68.69)	56.36 (45.58-58.97)	50.36 (47.08-61.52)	0.410
TOC (*μ*mol/L)^†^	7.77 (2.39-102.65)	6.36 (3.71-39.4)	7.69 (2.56-91.17)	14.31 (5.34-88.3)	0.534
TRC (GAE)^†^	0.11 (0.09-0.18)	0.14 (0.1-0.19)	0.17 (0.13-0.31)	0.14 (0.11-0.21)	0.074
TOC/TRC^†^	79.74 (14.61-596.81)	42.71 (16.27-305.42)	68.93 (10.32-795.19)	81.76 (21.35-609.38)	0.584
MDA/TRC^†^	30.19 ± 15.96	21.65 ± 11.09	26.64 ± 12.53	28.71 ± 17.19	0.152^2^
MDA/TOC^†^	0.34 (0.03-1.97)	0.41 (0.09-0.7)	0.52 (0.03-1.85)	0.31 (0.04-0.86)	0.899
AOPP/TRC^†^	1244.32 (919.53-1911.55)^b,c^	2.44 (1.43-2105.85)^b^	690.45 (387.05-1414.54)	912.11 (660.8-1239.08)	0.026
AOPP/TOC^†^	14.05 (1.59-80.08)	1241.82 (674.29-85.63)	16.21 (1.16-56.69)	8.01 (1.43-33.57)	0.305
NOx/CAT^†^	59.75 (42.65-74.17)	29.07 (3.66-74.72)	66.87 (56.66-73.14)	59.5 (53.17-70.06)	0.358
Albumin/TOC^†^	6.22 (0.44-20.78)	70.62 (56.22-12.67)	4.01 (0.33-16.51)	2.82 (0.51-8.57)	0.415
Albumin/TRC^†^	435.95 ± 250.94^b^	369.86 ± 179.31^b^	243.66 ± 177.67	313.12 ± 171.81	0.024^2^
TG/TBARS^†^	14.25 (11.44-22.71)^a,b^	11.19 (8.4-16.18)^c^	10.26 (7.47-13.2)^c^	15.49 (10.94-21.86)	0.002
TRC/albumin^†^	0.23^b^ (0.17-0.34)	0.27 (0.2-0.37)	0.35 (0.26-0.64)	0.28 (0.22-0.45)	0.05
AOPP/albumin^†^	2.56 (2.16-5.85)	2.81 (1.91-7.47)	2.43 (1.94-2.89)	2.31 (2.02-2.66)	0.319

^1^Kruskal-Wallis test; ^a^vs. cluster 2; ^b^vs. cluster 3; ^c^vs. cluster 4; ^†^median (25^th^–75^th^ percentile).

**Table 4:** Vitamin D and oxidative biomarker ratios in relation to cluster analysis.

Ratio^†^	Cluster 1	Cluster 2	Cluster 3	Cluster 4	*p* ^1^
Vitamin D/MPO	0.55 (0.41-0.75)	0.79^b,c^ (0.5-1.13)	0.49 (0.3-0.74)	0.40 (0.30-0.61)	0.011
Vitamin D/XO	2.04^a^ (1.67-2.82)	2.86^b,c^ (1.87-5.03)	1.77 (1.31-2.33)	1.62 (1.35-2.24)	0.001
Vitamin D/AOPP	0.43^a,c^ (0.2-0.52)	0.62 (0.22-0.86)	0.38 (0.2-0.51)	0.37 (0.21-0.5)	0.141
Vitamin D/MDA	17.41 (14.36-20.19)	29.67^b,c^ (22.59-40.11)	14.27 (8.83-20.3)	12.47 (8.65-18.16)	<0.001
Vitamin D/CAT	66.55 (56.36-72.11)	92.01^b,c^ (77.69-118.93)	58.39 (45.07-70.9)	59.47 (42.96-71.43)	<0.001
Vitamin D/NOx	1.18 (0.8-1.59)	1.41^c^ (1.08-1.86)	0.87 (0.65-1.29)	0.95 (0.74-1.26)	0.002
Vitamin D/TOC	5.95 (0.67-24.59)	12.2 (1.42-19.52)	5.36 (0.54-17.21)	2.93 (0.59-11.06)	0.081
Vitamin D/TRC	489.56^b,c^ (317.73-687.34)	599.49^b,c^ (411.29-809.45)	250.33 (148.98-373.19)	312.33 (224.67-539.03)	<0.001
Vitamin D/GGT	2.89^a,c^ (1.95-4.51)	4.72 (3.95-5.48)	2.61 (1.69-3.67)	2.01 (1.13-3.03)	<0.001
Vitamin D/albumin	1.21^a^ (0.99-1.3)	1.59 (1.39-2.14)	0.97^c^ (0.8-1.21)	1.03 (0.8-1.22)	<0.001

^1^Kruskal-Wallis test; ^a^vs. cluster 2; ^b^vs. cluster 3; ^c^vs. cluster 4; ^†^median (25^th^–75^th^ percentile).

## Data Availability

The data will be available upon reasonable request.
